# Characterization and Degradation Pathways of *Microbacterium resistens* MZT7, A Novel 17*β*-Estradiol-Degrading Bacterium

**DOI:** 10.3390/ijerph191711097

**Published:** 2022-09-05

**Authors:** Peng Hao, Sicheng Wu, Xiqing Zhang, Changlong Gou, Yuqiong Wang, Lixia Wang, Yanbin Zhu, Wangdui Basang, Yunhang Gao

**Affiliations:** 1 College of Animal Science and Technology, Jilin Agricultural University, Changchun 130118, China; 2 College of Animal Science and Technology, Inner Mongolia University for Nationalities, Tongliao 028000, China; 3 Northeast Institute of Geography and Agroecology, Chinese Academy of Sciences, Changchun 130102, China; 4 Institute of Animal Husbandry and Veterinary Medicine, Tibet Academy of Agriculture and Animal Husbandry Science, Lhasa 850009, China

**Keywords:** 17*β*-estradiol, *Microbacterium resistens* MZT7, biodegradation, metabolic pathway, genome analyses

## Abstract

Due to the ecotoxicity of 17β-estradiol (E2), residual E2 in the environment poses potential risks to human and animal health and ecosystems. Biodegradation is considered one of the most effective strategies to remove E2 from the environment. Here, a novel, efficient E2-degrading bacterial strain *Microbacterium resistens* MZT7 was isolated from activated sludge and characterized. The genome of strain MZT7 contained 4,011,347 bp nucleotides with 71.26% G + C content and 3785 coding genes. There was 86.7% transformation efficiency of 10 mg/L E2 by strain MZT7 after incubation for 5 d at optimal temperature (30 °C) and pH (7.0). This strain was highly tolerant to ranges in pH (5.0–11.0), temperature (20–40 °C), and salinity (2–8%). Adding sources of carbon (glucose, maltose, sucrose, or lactose) or nitrogen sources (urea, peptone, or beef extract) promoted the degradation of E2 by strain MZT7. However, when yeast extract was added as a nitrogen source, the degradation efficiency of E2 was inhibited. Metabolites were analyzed by LC-MS and three metabolic pathways of E2 degradation were proposed. Further, the intermediates dehydroepiandrosterone and androsta-1,4-diene-3,17-dione were detected, as well as identification of *kshB* and *fadD3* genes by KEGG, confirming one E2 degradation pathway. This study provided some insights into E2 biodegradation.

## 1. Introduction

It is well known that 17*β*-estradiol (E2) is a steroidal estrogen, mainly secreted by the adrenal cortex, testis, ovary, and placenta of humans and animals [1]. However, in most cases, it is not completely metabolized and is excreted into the environment [2]. Human and animal waste is considered to be the main source of steroidal estrogens in the environment [3]. Furthermore, accumulation of E2 in the environment has negative impacts on the ecosystem and on humans and animals [4,5]. In that regard, E2 can promote breast cancer in women [6,7], and induce feminization of male fish [8,9,10]. Therefore, there is an urgent need to mitigate E2 residues from the environment.

Studies have shown that physical methods such as membrane filtration and physical precipitation are inefficient in removing estrogens, while chemical methods may produce more toxic degradation products [11] Biodegradation is considered one of the most effective strategies to remove E2 from the environment. Microbial degradation has high efficiency, low cost, and environmental friendliness [12,13]. Many micro-organisms that can degrade E2 have been isolated, including *Sphingomonas* spp. [14,15], *Acinetobacter* spp. [16], *Stenotrophomonas maltophilia* [13,17], *Nitrosomonas* spp. [18], *Rhodococcus* spp. [14,19], and *Microbacterium* spp. [20]. However, most current research focuses on biological characteristics of functional bacteria, while little is known about the regulatory mechanisms and degradation genes involved in the degradation process [21,22,23]. To date, several possible estrogen biodegradation pathways have been proposed [11,14,21,23,24]. For example, it has been proposed that the E2 was dehydrogenized at C17 of ring D to yield estrone (E1) [25]. However, in contrast, Kurisu et al. reported that the E2 was directly cleaved via 4,5-seco pathway after C4 was hydroxylated [14]. Thus, it is suggested that bacteria in different taxa may employ multiple strategies to degrade estrogen [22].

Currently, genome sequencing combined with functional annotation is considered an effective approach to explore functional genes that encode enzymes involved in the biodegradation of environmental pollutants [26]. Moreover, among the dozens of E2-degrading strains available, only a few have been studied by whole-genome sequencing. In addition, among the known functional bacteria, there are apparently only limited studies on the degradation of E2 by *Microbacterium* spp. Yu et al. first suggested that *Microbacterium testaceum* KC5 degraded E2 to E1; however, no detailed studies on degradation of E2 by this genus were conducted [20]. And there are few reports on the degradation of E2 by this genus by analyzing the whole genome. *Microbacterium* spp. are widespread in the environment and capable of degrading a wide range of pollutants. Therefore, it is important to explore the mechanisms of E2 degradation by *Microbacterium* spp.

In this work, a new Gram-positive bacterium, *Microbacterium resistens* MZT7, was isolated from activated sludge and was identified as efficiently degrading E2. Objectives of this study were to: (1) determine the complete gene sequence of this strain and annotate and predict genes that may be involved in degradation; (2) determine degradation characteristics under various culture conditions; and (3) characterize metabolites by LC-MS analysis to reveal the E2 biodegradation pathways.

## 2. Materials and Methods

### 2.1. Chemicals and Cultures Medium

E2 (C_18_H_24_O_2_, MW = 272.38, >99% purity) was purchased from Solarbio Scientific Co., Ltd. (Beijing, China). HPLC grade of methanol (99.9% purity) and acetonitrile (99.95% purity) were obtained from Fisher Scientific Co., (Shanghai, China). All other chemicals were analytical grade. Estradiol was prepared as 1000 mg/L stock solution in methanol and stored at 4 °C in the dark.

Mineral salt medium (MSM, (NH_4_)_2_SO_4_ 1.0 g, K_2_HPO_4_ 1.0 g, KH_2_PO_4_ 0.5 g, MgSO_4_ 0.2 g, H_2_O 1000 mL, pH 7.0) was used for the enrichment of estrogen-degrading strains and degradation experiments. E2 enrichment or degradation medium is the addition of stock solutions to MSM to form various concentrations. The experiments were performed after complete evaporation of methanol. Luria-Bertani medium (LB, tryptone 10.0 g, NaCl 10 g, yeast extract 5.0 g, H_2_O 1000mL, pH 7.0) were used for normal strain culture. MSM and LB solid media were prepared by adding 15 g of agar powder per liter of media.

### 2.2. Enrichment, Isolation, and Identification of E2 Degrading Bacteria

Activated sludge samples were collected from manure storage tanks at a farm in Changchun, Jilin Province, China. A total of 10 g (wet weight) of sludge samples were inoculated in 100 mL of physiological saline and incubated in an oscillator at 30 °C for 12 h. Then, 1 mL of the samples were transferred to a 250 mL conical flask containing 100 mL of MSM (with 1 mg/L E2) and incubated in an oscillating incubator at 30 °C and 180 rpm for 7 d, with E2 as the sole carbon source. After five successive domestication processes at E2 concentrations of 1 to 40 mg/L, the final enrichment solution was diluted, plated onto 5 mg/L E2 MSM agar plates and incubated at 30 °C for 2–4 d. A strain with the highest degradability was detected by high-performance liquid chromatography (HPLC) and designated MZT7.

Morphological characteristics of the strain MZT7 were assessed by Gram staining and scanning electron microscopy, and then further identified by 16S rRNA gene sequencing. The total DNA of strain MZT7 was extracted and purified using Ezup Column Bacteria Genomic DNA Purification Kit (Shanghai, China). The 16S rDNA gene was amplified by PCR using universal primers 27F (5′-AGAGTT TGATCCTGGCTCAG-3′) and 1492R (5′-GGTTACCTTGTTACGACTT-3′). The PCR products were purified and sequenced by Sangon Biotech Co., Ltd. (Shanghai, China). The sequences were repeatedly aligned with other selected sequences from the same family strains from GenBank databases using ClustalW alignment. Software MEGA 7.0 was used to build a phylogenetic tree by neighbor-joining (NJ) methods.

### 2.3. Genome Sequencing

Strain *M. resistens* MZT7 was cultivated in LB liquid medium at 37 °C for 24 h, then bacteria were harvested by centrifugation at 8000 rpm for 15 min at 4 °C. Genomic DNA was extracted with the Sodium dodecylsulfate (SDS) method [27]. The harvested DNA was detected by the agarose gel electrophoresis and quantified by a Qubit^®^ 2.0 Fluorometer. The whole genome of stain MZT7 was sequenced using Nanopore PromethION platform and Illumina NovaSeq PE150 at the Beijing Novogene Bioinformatics Technology Co., Ltd., (Beijing, China). Glimmer software was used to predict the whole genome sequence of strain MZT7. Genes in the genome of strain MZT7 were predicted using GeneMarkS software and further annotated by the Nr, SwissProt, COG, KEGG, and GO databases. A circular genome map of strain MZT7 was generated using Circos software [28].

### 2.4. Biodegradation Characteristics of E2 by Strain MZT7

Prior to determining the effects of pH, temperature, salinity, inoculum, carbon, and nitrogen sources on biodegradation of E2 in MSM, strain MZT7 was grown in LB medium until exponential growth. After collection, cells were washed three times with sterile phosphate-buffered saline (PBS, pH 7.0), then resuspended and diluted to OD_600_ = 1.5. Degradation experiments were performed in 250 mL conical flasks with 100 mL of MSM with 10 mg/L E2, inoculated with 1% (*v*/*v*) bacterial suspension. The pH of the medium was adjusted to 3.0, 5.0, 7.0, 9.0, and 11.0 to investigate effects of pH on E2 degradation. To determine the effects of temperature on E2 degradation, the medium was incubated at 20, 25, 30, 35, and 40 °C. To investigate the effect of salinity on E2 degradation by isolating MZT7, NaCl was added to MSM at various mass fractions, including 0, 0.5, 1, 2, 4, and 8%. The inoculum levels of the strain MZT7 suspension were adjusted to 0.25, 0.5, 1.0, 2.0, and 4.0% to investigate the effect of inoculum level on E2 degradation. Effects of various sources of carbon (glucose, maltose, sucrose, and lactose) and nitrogen (urea, peptone, beef extract, and yeast extract) on the degradation of E2 (concentration, 10 mg/L) by strain MZT7 was further investigated. Each treatment was performed in triplicate, using treatments without strain MZT7 under the same conditions as a control. Flasks were incubated in a rotary shaker at 130 rpm and 30 °C and protected from light. Samples were taken on day 5 after incubation to determine E2 concentration and degradation efficiency. OD_600_ was measured after the degradation experiment to evaluate the effect of bacterial biomass on the degradation efficiency.

### 2.5. Determination of Residual E2

High-performance liquid chromatograph (HPLC, Shimadzu LC-2030 plus, Tokyo, Japan) system was used to detect E2; the E2 degradation efficiency of strain MZT7 was calculated based on residual E2 concentration in the medium. Briefly, 2 mL of culture medium was moved and added with an equal volume of acetonitrile, sonicated, and mixed evenly, then filtered through 0.22-μm filters (Jingteng, China). The settings of the HPLC/UV were as follows: A ZORBAX SB-C_18_ column (250 mm × 4.6 mm, 5 μm) was used for the separation and elution of E2 and its metabolites. The injection volume was 25 μL and the flow rate was set at 1 mL/min, with the mixture of acetonitrile and water (50/50, *v*/*v*) in the mobile phase. The column temperature was 30 °C, and the UV wavelength was 280 nm. The amount of E2 in the cultures was calculated by comparing the respective peak areas and the standard curves for each chemical, all with R^2^ values > 0.99.

### 2.6. Extraction and Detection of E2 and Its Metabolites

The E2 metabolites degraded by strain MZT7 were analyzed by the LC-MS (Liquid chromatography, Thermo U3000, Waltham, MA, USA; Mass Spectrometer, Thermo QE Focus, Waltham, MA, USA) system. First, 100 µL of each sample was put into 2 mL centrifuge tubes. Then, 400 µL of methanol (−20 °C) was added to each tube and vortexed for 60 s, then centrifuged at 4 °C for 10 min at 12,000 rpm, and all supernatant from each sample was transferred into another 2 mL centrifuge tube. Samples were dried in a vacuum, then dissolved in 150 µL of a mixture of 2-chlorophenylalanine (4 ppm) and methanol (20%:80%), and the supernatant filtered through a 0.22 µm membrane to obtain samples for LC-MS.

Chromatographic separation was used with an ACQUITY UPLC^®^ HSS T3 (150 × 2.1 mm, 1.8 µm, Waters, Milford, MA, USA) column maintained at 40 °C. The temperature of the autosampler was 8 °C. Gradient elution of analytes was carried out with 0.1% formic acid in water (C), and 0.1% formic acid in acetonitrile (D), or 5 mM ammonium formate in water (A), and acetonitrile (B) at a flow rate of 0.25 mL/min. Injection of 2 μL of each sample was done after equilibration. An increasing linear gradient of solvent B (*v*/*v*) was used as follows: 0~1 min, 2% B/D; 1~9 min, 2~50% B/D; 9~12 min, 50~98% B/D; 12~13.5 min, 98% B/D; 13.5~14 min, 98~2% B/D; 14~20 min, 2% Dpositive model (14~17 min, 2% B-negative model). The ESI-MSn experiments were used with the spray voltage of 3.5 kV and −2.5 kV in positive and negative modes, respectively. Sheath gas and auxiliary gas were set at 30 and 10 arbitrary units, respectively, and capillary temperature was 325 °C. The Orbitrap analyzer scanned over a mass range of *m*/*z* 81–1000 for full scan at a mass resolution of 70,000. Data-dependent acquisition (DDA) MS/MS experiments were performed with HCD scan. The normalized collision energy was 30 eV. Dynamic exclusion was implemented to remove unnecessary information in MS/MS spectra.

## 3. Results and Discussion

### 3.1. Isolation and Identification of Strain MZT7

**Figure 1:**
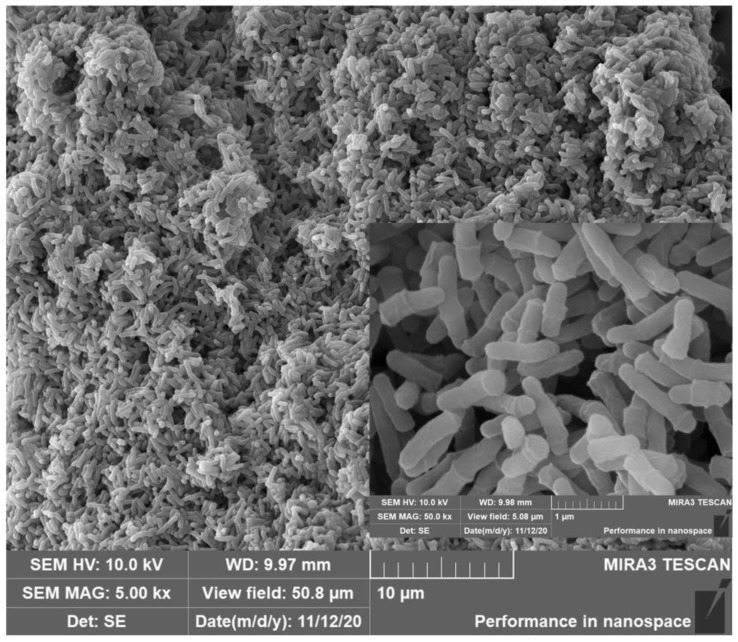
Scanning electron micrograph of strain MZT7.

**Figure 2:**
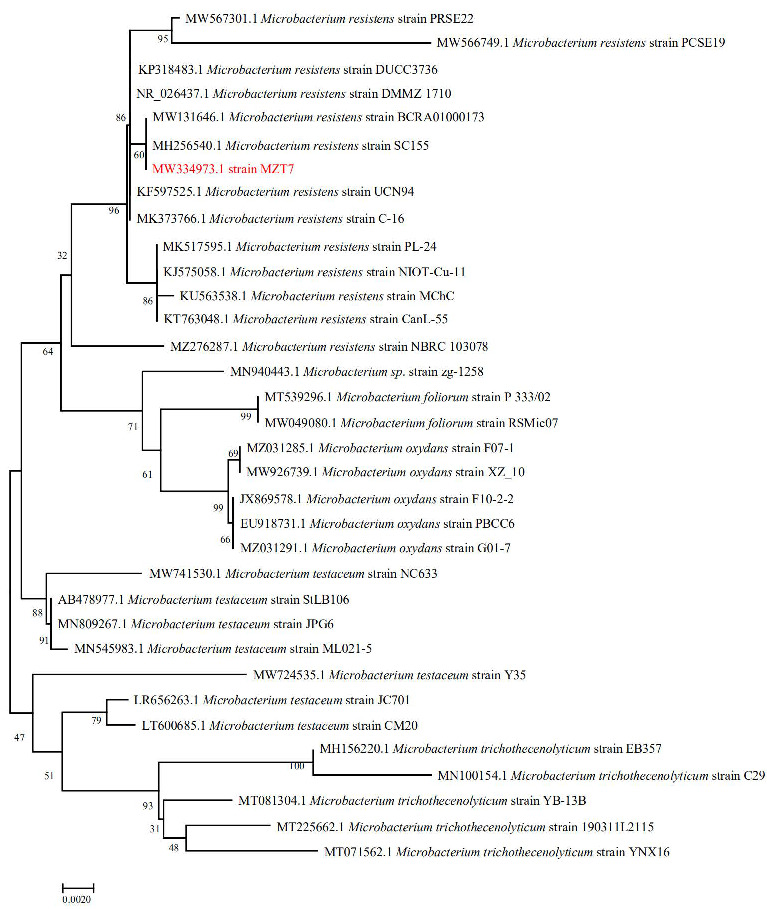
Phylogenetic tree based on the 16S rRNA sequence of strain MZT7 with a bootstrap value of 1000. The red label is the strain MZT7 isolated in this study.

A total of seven strains with E2 degradation ability were enriched and isolated from the activated sludge in the manure storage tank of the farm, of which, strain MZT7 was selected, due to its ability to efficiently degrade the E2. Strain MZT7 was a Gram-positive bacterium with a rod-like shape with a size of (0.25~0.4 μm) × (0.8~1.2 μm) (Figure 1 and Figure S1). The 16S rDNA sequence of strain MZT7 has been submitted to the NCBI database (accession number WM334973) (Table S1). Strain MZT7 was BLAST aligned in GenBank and was quite similar to some members of *Microbacterium* sp. Partial 16S rDNA sequencing of this isolate showed 99.93% similarity to *Microbacterium* sp. strain R2A9. From the phylogenetic tree based on the 16S rDNA gene sequence (Figure 2), it was close to *Microbacterium resistens.* Therefore, we concluded that isolate MZT7 was *Microbacterium resistens* (*M. resistens* MZT7).

### 3.2. Genome Characteristics and Database Analysis Related to E2 Degradation

The entire genome of strain MZT7 was sequenced and submitted to the GenBank database (accession no. CP082781); it was a complete circular chromosome of 4, 011,347 bp with a G + C content of 71.26% and no plasmid. In total 3785 coding DNA sequences (CDS) were predicted, which constituted 91.01% of the genome. In addition, the genome of strain MZT7 also encoded 46 tRNAs and six rRNAs. Among the annotated genes predicted by the whole genome, 3634, 2737, 2621, 3415, and 1256 genes were annotated by NR, COG, GO, KEGG, and Swiss-Prot, databases, respectively. The assembled complete genetic map was obtained (Figure S2).

**Figure 3:**
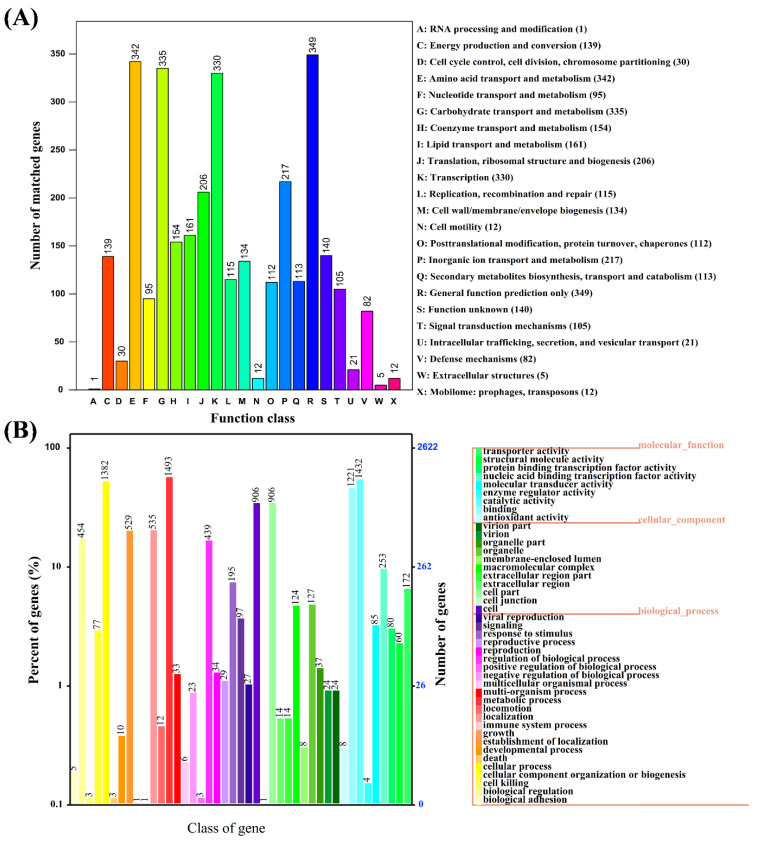
Functional annotation of strain MZT7. (**A**) COG function classification. (**B**) GO standard.

**Figure 4:**
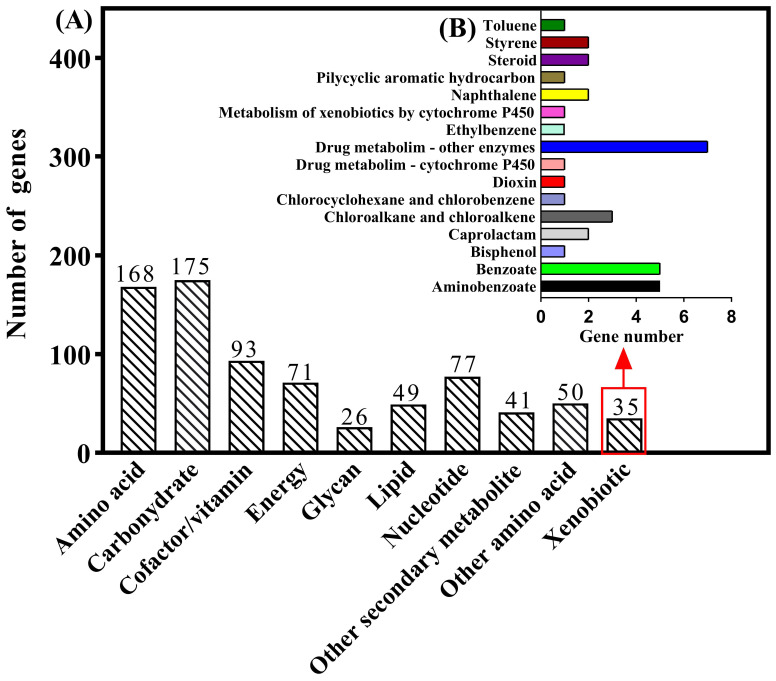
Metabolism of strain MZT7. (**A**) Genes distributed in the KEGG categories. (**B**) Genes involved in the KEGG pathways for xenobiotics biodegradation and metabolism.

The NR database is characterized by its comprehensive content and annotated results containing species information, which can be used for species classification. The predicted gene sequences of strain MZT7 were annotated by the NR database, as shown (Figure S3). Overall, 94.06% (3418/3634) of sequences matched with *Microbacterium* sp. and 83.68% (3041/3634) of sequences matched *Microbacterium resistents*, consistent with strain MZT7 being identified as a *Microbacterium resistents*. A total of 2737 genes had COG functional annotation (Figure 3A). Among them, protein functions of strain MZT7 were mainly focused on general function prediction only (R), amino-acid transport and metabolism (E), carbohydrate transport and metabolism (G), and transcription (K), with identification of 349, 342, 335, and 330 genes, respectively. The CDS of strain MZT7 were annotated in the GO database with a total of 2621 genes, of which 1432 were functional genes related to catalytic activity, 1493 were functional genes related to metabolic processes, and 172 genes with transport activity were annotated (Figure 3B). The 3785 coding genes of strain MZT7 were annotated to 3419 in the KEGG database, of which 1814 genes were annotated in the secondary functional classification. Functional genes were mainly concentrated on metabolism (Figure S4), with a total of 1299 genes annotated. Since strain degradation of E2 is a process of degrading foreign substances, this study focused on the database’s genetic annotation of xenobiotics biodegradation and metabolism. In total, 35 genes were identified in strain MZT7 as responsible for xenobiotic biodegradation and metabolism (Figure 4), including aminobenzoate, benzoate, bisphenol, caprolactam, dioxin, ethylbenzene, metabolism of xenobiotics, naphthalene, polycyclic aromatic hydrocarbon, steroid, styrene, and toluene. The genes annotated in each database that may be involved in E2 degradation are shown in Table S2.

It has been proposed that the E2 degradation pathway (i.e., the 4,5-seco pathway) starts with 17β-dehydrogenation to generate E1, which is performed by 17β-hydroxysteroid dehydrogenases (17-HSDs) [21,23,25]. Then, cytochrome P450 monooxygenase (CYP450) encoded by the *edcA* gene [23], or the *EstP* gene [21], catalyzes the 4-hydroxylation of E1 to generate 4-OH-E1. However, in *Sphingomonas* sp. KC8, that encodes a putative flavin-dependent monooxygenase (*OecB*) is considered to be the key enzyme for the hydroxylation of E1 [25]. In contrast, in the study of E2 degradation by *Novosphingobium* sp. ES2-1 flavin-dependent monooxygenase (*EstO*) is the key enzyme for further oxidation of 4-OH-E1 [21].

In conclusion, steroid dehydrogenase, flavin-dependent monooxygenase, CYP450 monooxygenase, catechol 1,2-dioxygenase, etc. are the key enzymes for the degradation of E2. These genes were annotated in the genome of strain MZT7. Short-chain dehydrogenases/reductases (SDR) are a large and functionally diverse family of enzymes that catalyze steroids, polycyclic aromatic hydrocarbons, and aliphatic aldehydes, with important roles in steroid degradation [29,30]. In addition, the KEGG database was annotated to 2 genes encoding genes involved in steroid degradation. They are 3-ketosteroid 9α-monooxygenase subunit B (*kshB*, EC 1.14.15.30, locus_tag=K8F61_04990) and HIP---CoA ligase (*fadD3*, EC 6.2.1.41, locus_tag=K8F61_15875). *kshB* is a monooxygenase widely present in steroid-degrading bacteria. It is essential to open the steroid B-ring, thereby initiating degradation of the steroid ring system [31,32]. In a recent study, Hsiao et al. demonstrated that *fadD3* gene expression was upregulated during the degradation of E1 by *Rhodococcus* sp. strain B50 [22]. Disruption of the gene *fadD3* led to a decrease in bacterial biomass and accumulation of the intermediate metabolite 3aα-H-4α(3′-propanoate)-7aβ-methylhexahydro-1,5-indanedione (HIP) [22]. It indicates that this gene plays an important role in the degradation of estrogen.

### 3.3. Effect of pH, Temperature, Salinity, and Inoculum Amount on E2 Biodegradation

**Figure 5:**
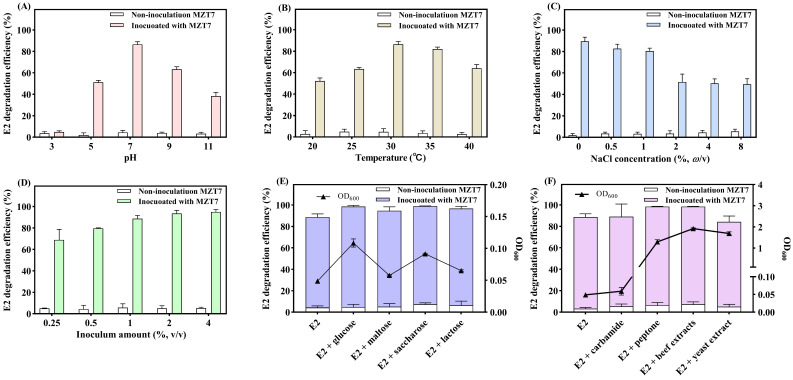
Effects of temperature (**A**), pH (**B**), salinity (**C**), inoculum amount (**D**), carbon (**E**), and nitrogen sources (**F**) on E2 degradation efficiency of 10 mg/L by strain MZT7.

The biodegradation ability of strain MZT7 against E2 under various environmental conditions was evaluated. At pH 7, strain MZT7 had the highest degradation efficiency (86.55%). As pH increased, degradation efficiency of E2 initially increased, then subsequently decreased (Figure 5A). There was high degradation activity in a wide pH range (5.0–11.0), with E2 degradation efficiency of 51.26 and 38.18% at pH 5 and 11, respectively. In general, pH is an important factor affecting intracellular transport and enzymatic activity of organic pollutants [33]. Based on current results, strain MZT7 can function in a broad range of acid–base environments.

Regarding temperature, E2 was efficiently degraded by strain MZT7 over a temperature range of 20–40 °C (Figure 5B), with degradation efficiency exceeding 50%. At the optimum degradation temperature of 30 °C, 86.47% of E2 at a concentration of 10 mg/L was removed after 5 d. Consequently, MZT7 is also well adapted to temperature. In addition, it is consistent with previous reports that E2-degrading bacteria prefer 30 °C to degrade E2 [29].

As the salinity increased to 10 mg/L, the degradation efficiency of E2 by strain MZT7 decreased from 89.61 to 80.48% (Figure 5C). Although the ability of MZT7 to degrade E2 was inhibited, it still maintained relatively high degradation efficiency. When the salinity of the culture was increased to 20, 40, and 80 mg/L, the degradation efficiency of E2 was significantly inhibited compared to the group without NaCl, but still remained at around 50%. Similar findings have been reported for other E2-degrading bacteria, such as *Stenotrophomonas maltophilia* SJTH1 [17]. This suggests that high salt concentrations lead to an osmotic pressure imbalance on the cell wall and loss of activity of functional micro-organisms.

The degradation efficiency of E2 by MZT7 was enhanced as there was an increase in bacterial suspension inoculum (Figure 5D), with a maximum degradation efficiency of 94.96% when the bacterial suspension inoculum of MZT7 was 4%. In addition, E2 degradation efficiency were 68.90, 79.69, 88.69, and 93.57% at inoculum levels of 0.25, 0.5, 1.0, and 2.0%, respectively. Degradation of niclosulfuron by *Serratia marcescens* N80 was 73.9 and 92.9% at 2 and 3% inoculum, respectively. However, the degradation efficiency decreased when the inoculum of the strain was ≥3% [34]. This may be because when the initial energy is constant, excessive inoculum leads to poor bacterial growth, resulting in a decrease in the degradation efficiency of organic matter.

### 3.4. Effect of Carbon Source and Nitrogen Source on E2 Biodegradation

Four single-carbon sources (glucose, maltose, sucrose, or lactose) were added to MSM in the presence of 10 mg/L E2 to investigate effects of strain MZT7 on E2 degradation. The addition of 0.1% glucose, maltose, sucrose, and lactose increased the degradation efficiency of strain MZT7 on E2 at the same degradation time compared to the control treatment without additional carbon sources (Figure 5E). Among them, the addition of sucrose maximized degradation efficiency (99.01%). Similar results have been reported for the biodegradation of other organic pollutants, where addition of sodium lactate accelerated degradation of bensulfuron-methyl by strain *Brevibacterium* sp. [35]. Furthermore, biodegradation of carbendazim by strain *Rhodococcus* sp. CX-1 was also enhanced by the addition of 100 mg/L glucose, fructose, or sucrose [36]. *Pseudomonas aeruginosa* DDMZ1-2 significantly up-regulated the reductase gene *azoR3* upon addition of fructose, which accelerated biodegradation of the azo dye [37]. Urea, peptone, and beef extract increased degradation of E2 compared to the blank treatment group (Figure 5F), whereas addition of yeast extract slightly reduced the degradation efficiency. In previous studies, the addition of nitrogen sources promoted degradation of contaminants. For example, exogenous nitrogen addition accelerated degradation of tetrabromobisphenol A, with organic nitrogen sources generally superior to inorganic nitrogen [38]. In this study, additional carbon and nitrogen sources promoted the degradation of E2 by strain MZT7. As shown in Figure 5E,F, the increase in the biomass of the strains in the system was responsible for the improved degradation efficiency. The addition of yeast extract increased the biomass but slightly inhibited degradation, probably because the strain preferentially utilized yeast extract over E2.

Co-metabolism is the process by which organic compounds are biodegraded in the presence of a single energy substance [39]. Many reports have demonstrated that co-substrates promote degradation of a variety of refractory substances, and that co-substrates accelerated redox reactions, up-regulated expression of functional enzymes, and accelerated biodegradation and mineralization of contaminants [40,41]. Additional carbon and nitrogen sources can provide abundant energy to meet the needs of microbial growth [39]. However, providing additional carbon and nitrogen sources did not definitely promote degradation of pollutants. In this experiment, the addition of yeast extract reduced the degradation efficiency of strain MZT7 to E2, which may be the strain that preferentially utilizes yeast extract. It is also possible that the yeast extract competes with E2. Zhang et al. also reported that the addition of carbon sources seemed to inhibit the degradation of E2, and concluded that strain FJ1 preferred the use of additional carbon sources over E2 [11]. There is also evidence that the efficiency of degradation of pollutants by degrading bacteria is influenced by the concentration of added carbon sources [24]. In the future, the mechanism of E2 degradation by strain MZT7 needs to be further studied.

### 3.5. Biodegradation Products and Metabolic Pathways

The degradation products of E2 by strain MZT7 were detected by LC-MS/MS, and degradation pathways were speculated. After biodegradation of E2 by strain MZT7, six E2 metabolites were identified in the degradation solution (Table 1, Figures S5 and S6). At a retention time of 13.07 min, the substance P1 was identified as estrone (E1). In almost all studies on microbial degradation of E2, E1 has been detected as an intermediate metabolite [14,17,19,20,24,42], and it is the main degradation product of E2 [2,4]. Moreover, many studies suggest that microbial degradation of E2 begins first with the D-ring, where the hydroxyl group at C-17 is oxidized to a ketone group and E2 is converted to E1 [25,29]. Intermediate P2 was identified as estriol (E3), and some micro-organisms can metabolize E1 to E3 [29,43,44]. Intermediates P3 and P4 were also identified as Homovanillic acid and Vanillylmandelic acid at m/z 182.06 and 167.01, respectively. Products P5-P6 were identified as Dehydroepiandrosterone and Androsta-1,4-diene-3,17-dione at m/z 269.2 and 284.18, respectively, and they were enriched in steroid hormone biosynthesis and steroid degradation pathways. These metabolites of E2, P3-P6, have apparently not been reported in previous biodegradation studies.

**Figure 6:**
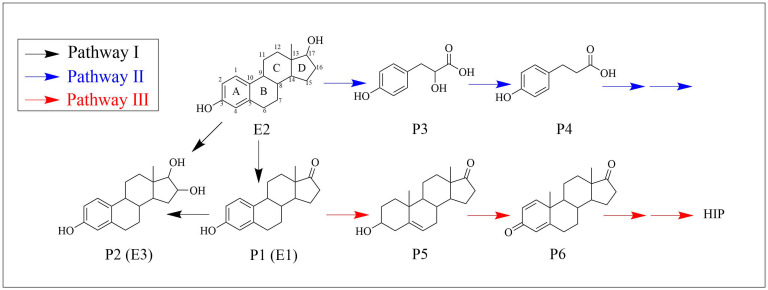
Possible pathways for 17β-estradiol degradation by strain MZT7.

Three potential metabolic pathways of E2 were proposed (Figure 6), based on reported degradation pathways and the products detected in this study. In pathway I, E1 was produced by dehydrogenation of E2 at C-17 of D-ring, and E3 was obtained by conversion of E1 and E2. A similar metabolic mechanism was reported in several biodegradable E2 studies [29,44]. Previous studies have shown that E1, E2, and E3 can be transformed into each other under certain conditions [4]. In Pathway II, E2 was metabolized to P3 and then P3 was metabolized to P4. Substances similar in structure to P3 have been reported [14]. In their study of the metabolites of E2 after degradation by *Sphingomonas* sp. ED8, Kurisu et al. [14] identified the fragment profile to be identical to the fragment profile of 3-(4-hydroxyphenyl)-2-hydroxyprop-2-enoic acid in the NIST library. The C8–C9 of this substance are connected by a double bond, which is a single bond in our study. It can be seen that in order to degrade E2, the micro-organisms attack other saturated rings (B–D rings) in addition to the A-ring. For example, the *Ochrobactrum* sp. strain FJ1 first hydroxylated the A-ring of E2, and then cleaved the B and C-rings [11]. Regarding Path III, after E2 was degraded into E1, E1 was further degraded into P5, and then P5 to P6. As mentioned earlier, substances P5 and P6 in this metabolic pathway are enriched in the steroid degradation pathway which eventually goes to the HIP degradation pathway. Both estrogen and androgen catabolic pathways have been reported to converge at HIP [45]. Wu et al. investigated degradation of E2 by *Sphingomonas* sp. strain KC8, detected a common steroid metabolite, HIP, in bacterial cultures, and suggested it was generated by oxidation of the product after cleavage of the A/B-ring of E2 [46]. In this study, although HIP was not observed, its upstream material was detected, indicating that E2 enters this degradation pathway under the biological action of strain MZT7.

### 3.6. Genome Annotation Reveals Biodegradation Characteristics and Pathways

The high degradation efficiency of E2 by strain MZT7 was attributed to the abundance of transporter proteins in its genome (Figure 3 and Figure S4). Xu et al. reported that the ABC transporter is involved in the uptake and transport of steroids [47]. Further, strain MZT7 has good tolerance to complex environments, including pH (5.0–11.0), temperature (20–40 °C), and salinity (8%). Based on mining of genomic data, strain MZT7 encoded at least 29 genes that responded to various stresses, e.g., pH, cold/heat shock, and osmotic stress (Table S3). These genes, such as CspA, DnaJ/DnaK, ClpB, and OsmC, were reported to contribute to resistance to cold shock, heat shock, osmotic stress, and extreme pH [48,49,50,51]. These findings accounted for the ability of the strain to degrade E2 under unfavorable environments and also indicated its potential to be used in complex environments.

Based on LC-MS analysis, we inferred that strain MZT7 catabolized E2 to E1. 17*β*-hydroxysteroid dehydrogenase (17*β*-HSDs) has a corresponding function in this process [19,52,53]. In addition, it was reported that 3-oxoacyl-(acyl-Carrier-protein) reductase has a function similar to 17*β*-HSDs [54]. In this study, although the genome-wide annotation results of strain MZT7 were not directly annotated to 17*β*-HSDs, by analyzing the genome annotations, we mined several short-chain dehydrogenases in the NR, GO, COG, and KEGG databases, respectively (Table S2), and E2 can be dehydrogenated to E1 by these encoded proteins. Following generation of E1, Chen et al. reported that E1 was catalyzed by 4-hydroxylase to generate 4-hydroxyestrone (4-OH-E1), and then the ring A of 4-OH-E1 was cleaved by 4,5-dioxygenase [25]. The genes encoding these functions were found in the genome of strain MZT7, such as CYP450 (locus_tag=K8F61_09760), and Flavin-dependent oxidoreductase (locus_tag=K8F61_02060, locus_tag=K8F61_02685, etc.). In this study, no products related to the cleavage of ring A were detected. In contrast, products of B-D ring cleavage were observed, e.g., substances P3 and P4, but the molecular mechanism of this reaction was not clear. In addition, the coding genes *kshB* and *fadD3* were enriched in the KEGG steroid degradation pathway, and concurrently, dehydroepiandrosterone (P5) and androsta-1,4-diene-3,17-dione (P6) in the pathway were detected by LC-MS. These results were regarded as evidence that E2 enters the steroid degradation pathway under the biological action of strain MZT7.

## 4. Conclusions

In this study, an E2-degrading strain was isolated from farm-derived activated sludge and identified as *Microbacterium resistens* MZT7. The genome of strain MZT7 contained a chromosome of 4, 011,347 bp with a GC content of 71.26%. Strain MZT7 had good resistance to complex environments, including pH (5.0–11.0), temperature (20–40 °C), and salinity (2–8%). Addition of various carbon sources (glucose, maltose, sucrose, or lactose) and nitrogen sources (urea, peptone, or beef extract) promoted degradation of E2 by MZT7, although yeast extract was inhibitory. The main E2 degradation products were E1, E3, and dehydroepiandrosterone, and three possible E2 degradation pathways were proposed. The genome annotation results indicated that the strain possessed abundant transporter proteins, resistance-related encoded proteins, and potential degradation genes. Further, related genes in the steroid degradation pathway were also annotated. Therefore, strain MZT7 metabolized E2 via the steroid degradation pathway. These findings provided a new profile for the biodegradation of E2. Future studies are needed to examine the function of degradation genes through transcriptomic analysis, RT-qPCR, and gene expression assays to refine the E2 degradation mechanism.

## Figures and Tables

**Table 1:** LC-MS analysis of metabolites involved in E2 degradation by strain MZT7.

ID	Formula	RT ^a^ (min)	*m*/*z* ^b^	Exact Mass
E2	C_18_H_24_O_2_	12.52	273.2530	272.1776
P1 (E1)	C_18_H_22_O_2_	13.07	271.1686	270.162
P2 (E3)	C_18_H_24_O_3_	9.12	288.1493	288.1725
P3	C_9_H_10_O_4_	13.03	182.0579	182.0579
P4	C_9_H_10_O	1.22	167.0126	166.063
P5	C_19_H_28_O_2_	13.49	269.2105	288.2089
P6	C_19_H_24_O_2_	7.81	284.1853	284.1776

^a^ RT, retention time; ^b^ mass-to-charge ratio.

## Data Availability

The 16S rDNA sequence for the isolate MZT7 has been uploaded to NCBI with accession number of MW334973. The whole genome shotgun project has been deposited at NCBI with accession number of CP082781 (submission ID SUB10297363, BioProject ID PRJNA759364, and BioSample ID SAMN21165868).

## Supplementary Materials

The following supporting information can be downloaded at: https://www.mdpi.com/article/10.3390/ijerph191711097/s1, Figure S1 Morphological observation of strain MZT7. (A) Colony morphology, (B) Gram stain picture. Figure S2. The circular map of the strain MZT7 genome DNA. Figure S3 NR database species annotation statistics. Figure S4 KEGG pathway annotation. Figure S5 Base peak chromatogram. (A) Negative Mode, (B) Positive Mode. Figure S6 Mass spectra and the proposed structures of E2 metabolites; Table S1 The 16S rRNA gene sequence of strain MZT7. Table S2 Potential E2 degradation genes in the genome of strain MZT7. Table S3 Stress-related genes in the genome of strain MZT7.
