# Supplementary material for: Characterization and Degradation Pathways of Microbacterium resistens MZT7, A Novel 17β-Estradiol-Degrading Bacterium
Source: Int J Environ Res Public Health. 2022 Sep 5;19(17):11097. doi: 10.3390/ijerph191711097 (PMC9518027; doi:10.3390/ijerph191711097)

Supplementary Materials

Characterization and degradation pathway of a newly isolated 17β-estradiol degrading bacterium *Microbacterium resistens* MZT7.

Peng Hao[a], Sicheng Wu[a], Xiqing Zhang[a], Changlong Gou[b], Yuqiong Wang[b], Lixia Wang[c], Yanbin Zhu[d], Wangdui Basang[d], Yunhang Gao[a *]

[a]*College of Animal Science and Technology, Jilin Agricultural University, Changchun 130118, China.*
[b]*College of Animal Science and Technology, Inner Mongolia University for Nationalities, Tongliao, Inner Mongolia, 028000, China.*
[c]*Northeast Institute of Geography and Agroecology, Chinese Academy of Sciences, Changchun 130102, China.*
[d]*Institute of Animal Husbandry and Veterinary Medicine, Tibet Academy of Agriculture and Animal Husbandry Science, Lhasa 850009, China*
*Corresponding authors: Tel.: +86 13159752912
Email: gaoyunhang@163.com (Yunhang Gao).

Total number of pages: 13; Total number of Tables: 3; Total number of Figures: 6

Table legends

Table S1 The 16S rRNA gene sequence of strain MZT7.
Table S2 Potential E2 degradation genes in the genome of strain MZT7.
Table S3 Stress-related genes in the genome of strain MZT7.

Figure Captions

Figure S1 Morphological observation of strain MZT7. (A) Colony morphology, (B) Gram stain picture.
Figure S2. The circular map of the strain MZT7 genome DNA
Figure S3 NR database species annotation statistics.
Figure S4 KEGG pathway annotation.
Figure S5 Base peak chromatogram. (A) Negative Mode, (B) Positive Mode.
Figure S6 Mass spectra and the proposed structures of E2 metabolites.

Table S1. The 16S rRNA gene sequence of strain MZT7.

16S rDNA size (bp)	1379
Similarity	100 %
Accession number	WM334973
Putative genus	*Microbacterium*
**16S rRNA gene sequence:**<br>CAAGGGTTAGGCCACCGGCTTCAGGTGTTACCGACTTTCATGACTTGACGGGCGGT<br>GTGTACAAGACCCGGGAACGTATTCACCGCAGCGTTGCTGATCTGCGATTACTAGC<br>GACTCCGACTTCATGAGGTCGAGTTGCAGACCTCAATCCGAACTGGGACCGGCTTT<br>TTGGGATTCGCTCCACCTCACGGTATTGCAGCCCTTTGTACCGGCCATTGTAGCATGC<br>GTGAAGCCCAAGACATAAGGGGCATGATGATTTGACGTCATCCCCACCTTCCTCCG<br>AGTTGACCCCGGCAGTATCCCATGAGTTCCCACCATAACGTGCTGGCAACATAGAA<br>CGAGGGTTGCGCTCGTTGCGGGACTTAACCCAACATCTCACGACACGAGCTGACGA<br>CAACCATGCACCACCTGTTCACGAGTGTCCAAAGAGTTGACCATTTCTGGCCCGTT<br>CTCGTGTATGTCAAGCCTTGGTAAGGTTCTTCGCGTTGCATCGAATTAATCCGCATGC<br>TCCGCCGCTTGTGCGGGTCCCCGTCAATTCCTTTGAGTTTTAGCCTTGCGGCCGTACT<br>CCCCAGGCGGGGAACTTAATGCGTTAGCTGCGTCACGGAATCCGTGGAAAGGACC<br>CCACAACTAGTTCCCAACGTTTACGGGGTGGACTACCAGGGTATCTAAGCCTGTTTG<br>CTCCCCACCCTTTCGCTCCTCAGCGTCAGTTACGGCCCAGAGATCTGCCTTCGCCAT<br>CGGTGTTCCTCCTGATATCTGCGCATTCCACCGCTACACCAGGAATTCCAATCTCCC<br>CTACCGCACTCTAGTCTGCCCGTACCCACTGCAGGCCCGAGGTTGAGCCTCGGGAT<br>TTCACAGCAGACGCGACAAACCGCCTACGAGCTCTTTACGCCCAATAATTCCGGAT<br>AACGCTTGCGCCCTACGTATTACCGCGGCTGCTGGCACGTAGTTAGCCGGCGCTTTT<br>TCTGCAGGTACCGTCACTTTCGCTTCTTCCCTGCTAAAAGAGGTTTACAACCCGAAG<br>GCCGTCATCCCTCACGCGGCGTTGCTGCATCAGGCTTGCGCCCATTGTGCAATATTC<br>CCCACTGCTGCCTCCCGTAGGAGTCTGGGCCGTGTCTCAGTCCCAGTGTGGCCGGT<br>CACCCTCTCAGGCCGGCTACCCGTCGACGCCTTGGTGAGCCATTACCTCACCAACA<br>AGCTGATAGGCCGCGAGCCCATCCCAGACCGAAAAATCTTTCCAAACGTTGACCAT<br>GCGGCCACGTCTCGTATCCAGTATTAGACGCCGTTTCCAGCGCTTATCCCAGAGTCC<br>AGGGCAGGTTGCTCACGTGTTACTCACCCGTTCGCCACTAATCCACCAGAGCAAGC<br>CCCGGCTTCATCGTTCGACTGCA	

Table S2. Potential E2 degradation genes in the genome of strain MZT7

Databases	Types	Enzymes	Encoding gene ID	Number
COG	dehydrogenase	NAD(P)-dependent dehydrogenase, short-chain alcohol dehydrogenase family	locus_tag=K8F61_00480, locus_tag=K8F61_00785, locus_tag=K8F61_01190, locus_tag=K8F61_02075, locus_tag=K8F61_03240, locus_tag=K8F61_03260, locus_tag=K8F61_03825, locus_tag=K8F61_04120, locus_tag=K8F61_04615, locus_tag=K8F61_04975, locus_tag=K8F61_05480, locus_tag=K8F61_07240, locus_tag=K8F61_07450, locus_tag=K8F61_08345, locus_tag=K8F61_08500, locus_tag=K8F61_08505, locus_tag=K8F61_09625, locus_tag=K8F61_10370, locus_tag=K8F61_10505, locus_tag=K8F61_10970, locus_tag=K8F61_12535, locus_tag=K8F61_12545, locus_tag=K8F61_12555, locus_tag=K8F61_14450, locus_tag=K8F61_15315, locus_tag=K8F61_15320, locus_tag=K8F61_15540, locus_tag=K8F61_15790, locus_tag=K8F61_15855, locus_tag=K8F61_15860, locus_tag=K8F61_15870, locus_tag=K8F61_15895, locus_tag=K8F61_15910, locus_tag=K8F61_15940, locus_tag=K8F61_15970	35
		Short-chain dehydrogenase	locus_tag=K8F61_03015, locus_tag=K8F61_06875, locus_tag=K8F61_10835, locus_tag=K8F61_16890	4
		3-hydroxyisobutyrate dehydrogenase or related beta-hydroxyacid dehydrogenase	locus_tag=K8F61_00230, locus_tag=K8F61_02220, locus_tag=K8F61_15720, locus_tag=K8F61_16030, locus_tag=K8F61_18225	5
		FAD/FMN-containing dehydrogenase	locus_tag=K8F61_00725, locus_tag=K8F61_00925, locus_tag=K8F61_08820, locus_tag=K8F61_14505	4
		Acyl-CoA reductase or other NAD-dependent aldehyde dehydrogenase	locus_tag=K8F61_02215, locus_tag=K8F61_02860, locus_tag=K8F61_02865, locus_tag=K8F61_06310, locus_tag=K8F61_08195, locus_tag=K8F61_08590, locus_tag=K8F61_08660, locus_tag=K8F61_09115, locus_tag=K8F61_09120, locus_tag=K8F61_11310	17

			locus_tag=K8F61_13770, locus_tag=K8F61_15395, locus_tag=K8F61_15710, locus_tag=K8F61_16080, locus_tag=K8F61_16210, locus_tag=K8F61_16245, locus_tag=K8F61_16270	
		Acyl-CoA dehydrogenase related to the alkylation response protein AidB	locus_tag=K8F61_03920, locus_tag=K8F61_06315, locus_tag=K8F61_08580, locus_tag=K8F61_08960, locus_tag=K8F61_11500, locus_tag=K8F61_11570, locus_tag=K8F61_12030, locus_tag=K8F61_15715, locus_tag=K8F61_15915, locus_tag=K8F61_15920, locus_tag=K8F61_15925, locus_tag=K8F61_15935	12
	hydroxylases	2-polyprenyl-6-methoxyphenol hydroxylase and related FAD-dependent oxidoreductases	locus_tag=K8F61_02355, locus_tag=K8F61_03100, locus_tag=K8F61_03135, locus_tag=K8F61_08640, locus_tag=K8F61_10975, locus_tag=K8F61_13850, locus_tag=K8F61_14490	7
	oxygenase	Flavin-dependent oxidoreductase, luciferase family	locus_tag=K8F61_02060, locus_tag=K8F61_02685, locus_tag=K8F61_04210, locus_tag=K8F61_05470, locus_tag=K8F61_06985, locus_tag=K8F61_10570, locus_tag=K8F61_13690, locus_tag=K8F61_16395	8
		Catechol 2,3-dioxygenase or other lactoylglutathione lyase family enzyme	locus_tag=K8F61_00360, locus_tag=K8F61_02700, locus_tag=K8F61_03060, locus_tag=K8F61_04135, locus_tag=K8F61_08670, locus_tag=K8F61_09075, locus_tag=K8F61_09410, locus_tag=K8F61_09830, locus_tag=K8F61_09845, locus_tag=K8F61_10325, locus_tag=K8F61_11315, locus_tag=K8F61_12690, locus_tag=K8F61_13605, locus_tag=K8F61_13935, locus_tag=K8F61_15080	15
		NAD(P)H-dependent flavin oxidoreductase YrpB	locus_tag=K8F61_15950, locus_tag=K8F61_16745	2
	CYP450	Cytochrome P450	locus_tag=K8F61_09760	1
KEGG	dehydrogenase	3alpha(or 20beta)-hydroxysteroid dehydrogenase	locus_tag=K8F61_02075, locus_tag=K8F61_03240, locus_tag=K8F61_07450, locus_tag=K8F61_08345, locus_tag=K8F61_15910	5
	oxygenase	3-ketosteroid 9alpha-monooxygenase subunit B	locus_tag=K8F61_04990	1
		heme oxygenase	locus_tag=K8F61_09760	1

	-	HIP---CoA ligase	locus_tag=K8F61_15875	1
NR	dehydrogenase	short-chain dehydrogenase	locus_tag=K8F61_00480, locus_tag=K8F61_03015, locus_tag=K8F61_03260, locus_tag=K8F61_04120, locus_tag=K8F61_06875, locus_tag=K8F61_09625, locus_tag=K8F61_10370, locus_tag=K8F61_10505, locus_tag=K8F61_10775, locus_tag=K8F61_10970, locus_tag=K8F61_12535, locus_tag=K8F61_15320, locus_tag=K8F61_15540, locus_tag=K8F61_15855, locus_tag=K8F61_15860, locus_tag=K8F61_15940, locus_tag=K8F61_15970	17
		NADH dehydrogenase	locus_tag=K8F61_00890	1
		3-alpha-hydroxysteroid dehydrogenase	locus_tag=K8F61_02075, locus_tag=K8F61_03240, locus_tag=K8F61_07450, locus_tag=K8F61_08345, locus_tag=K8F61_15910	5
		3-beta-hydroxysteroid dehydrogenase	locus_tag=K8F61_18360	1
		alcohol dehydrogenase	locus_tag=K8F61_03480, locus_tag=K8F61_07770, locus_tag=K8F61_08365, locus_tag=K8F61_11475, locus_tag=K8F61_11785, locus_tag=K8F61_16890	6
		acyl-CoA dehydrogenase	locus_tag=K8F61_03920, locus_tag=K8F61_04675, locus_tag=K8F61_06315, locus_tag=K8F61_07735, locus_tag=K8F61_08580, locus_tag=K8F61_08960, locus_tag=K8F61_11500, locus_tag=K8F61_11570, locus_tag=K8F61_12030, locus_tag=K8F61_15715, locus_tag=K8F61_15920, locus_tag=K8F61_15925, locus_tag=K8F61_15935	13
	oxygenase	monooxygenase	locus_tag=K8F61_03135, locus_tag=K8F61_08350, locus_tag=K8F61_08640, locus_tag=K8F61_10975, locus_tag=K8F61_13690, locus_tag=K8F61_15700, locus_tag=K8F61_15775	6
		heme oxygenase	locus_tag=K8F61_14170	1
	CYP450	Cytochrome P450	locus_tag=K8F61_09760	1

GO	dehydrogenase	3-beta-hydroxy-delta5-steroid dehydrogenase activity	locus_tag=K8F61_00480, locus_tag=K8F61_07950, locus_tag=K8F61_08505, locus_tag=K8F61_10775, locus_tag=K8F61_13095, locus_tag=K8F61_16280, locus_tag=K8F61_18880	7
		3-hydroxyacyl-CoA dehydrogenase activity	locus_tag=K8F61_00760, locus_tag=K8F61_01940, locus_tag=K8F61_02355, locus_tag=K8F61_02360, locus_tag=K8F61_03095, locus_tag=K8F61_03135, locus_tag=K8F61_03175, locus_tag=K8F61_04265, locus_tag=K8F61_04675, locus_tag=K8F61_04805, locus_tag=K8F61_04945, locus_tag=K8F61_04975, locus_tag=K8F61_06080, locus_tag=K8F61_06535, locus_tag=K8F61_07240, locus_tag=K8F61_07930, locus_tag=K8F61_08120, locus_tag=K8F61_08500, locus_tag=K8F61_08505, locus_tag=K8F61_08585, locus_tag=K8F61_09270, locus_tag=K8F61_10370, locus_tag=K8F61_10610, locus_tag=K8F61_12925, locus_tag=K8F61_13115, locus_tag=K8F61_14375, locus_tag=K8F61_14770, locus_tag=K8F61_15540, locus_tag=K8F61_15720, locus_tag=K8F61_15870, locus_tag=K8F61_16720, locus_tag=K8F61_16730, locus_tag=K8F61_17490, locus_tag=K8F61_17940, locus_tag=K8F61_18160, locus_tag=K8F61_18225, locus_tag=K8F61_18710, locus_tag=K8F61_18880	38
		acyl-CoA dehydrogenase	locus_tag=K8F61_03920, locus_tag=K8F61_04690, locus_tag=K8F61_06315, locus_tag=K8F61_08580, locus_tag=K8F61_08960, locus_tag=K8F61_11500, locus_tag=K8F61_11570, locus_tag=K8F61_12025, locus_tag=K8F61_12030, locus_tag=K8F61_15710, locus_tag=K8F61_15715, locus_tag=K8F61_15925, locus_tag=K8F61_15935, locus_tag=K8F61_16245, locus_tag=K8F61_16270	15
	oxygenase	flavin-containing monooxygenase activity	locus_tag=K8F61_00265, locus_tag=K8F61_03285, locus_tag=K8F61_04265, locus_tag=K8F61_04945, locus_tag=K8F61_05000, locus_tag=K8F61_06585, locus_tag=K8F61_08350, locus_tag=K8F61_13115, locus_tag=K8F61_15195, locus_tag=K8F61_15775, locus_tag=K8F61_18710	11

Table S3. Stress-related genes in the genome of strain MZT7.

	Ko_name	Ko_defi	Gene_id
Cold shock	cspA	cold shock protein (beta-ribbon, CspA family)	locus_tag=K8F61_18980
Heat shock	rph	ribonuclease PH (EC 2.7.7.56)	locus_tag=K8F61_01595
	clpX, CLPX	ATP-dependent Clp protease ATP-binding subunit ClpX	locus_tag=K8F61_02870
	hrcA	heat-inducible transcriptional repressor	locus_tag=K8F61_03440
	dnaJ	molecular chaperone DnaJ	locus_tag=K8F61_03445, locus_tag=K8F61_14685
	clpS	ATP-dependent Clp protease adaptor protein ClpS	locus_tag=K8F61_05675
	clpP	ATP-dependent Clp protease, protease subunit	locus_tag=K8F61_06785, locus_tag=K8F61_06790
	HSP90A, htpG	molecular chaperone HtpG	locus_tag=K8F61_09095
	dnaK, HSPA9	molecular chaperone DnaK	locus_tag=K8F61_14675
	GRPE	molecular chaperone GrpE	locus_tag=K8F61_14680
	hspR	heat shock protein HspR	locus_tag=K8F61_14690
	clpB	ATP-dependent Clp protease ATP-binding subunit ClpB	locus_tag=K8F61_14865
	clpC	ATP-dependent Clp protease ATP-binding subunit ClpC	locus_tag=K8F61_16425
	groES, HSPE1	chaperonin GroES	locus_tag=K8F61_17835
Osmotic stress	osmC	osmotically inducible protein OsmC	locus_tag=K8F61_06900
	aqpZ	Aquaporin Z	locus_tag=K8F61_09290, locus_tag=K8F61_13320
	opuBD	osmoprotectant transport system permease protein	locus_tag=K8F61_12905, locus_tag=K8F61_12910
	opuA	osmoprotectant transport system ATP-binding protein	locus_tag=K8F61_12915
	proP	MFS transporter, MHS family, proline/betaine transporter	locus_tag=K8F61_07235, locus_tag=K8F61_11980
PH	ATPF1B, atpD	F-type H+-transporting ATPase subunit beta	locus_tag=K8F61_07310
	ATPF1E, atpC	F-type H+-transporting ATPase subunit epsilon	locus_tag=K8F61_07305
	ATPF1G, atpG	F-type H+-transporting ATPase subunit gamma	locus_tag=K8F61_07315

ATPF0C, atpE	F-type H+-transporting ATPase subunit c	locus_tag=K8F61_07335
ATPF0A, atpB	F-type H+-transporting ATPase subunit a	locus_tag=K8F61_07340
nhaA	Na+:H+ antiporter, NhaA family	locus_tag=K8F61_03230

Figure S1. Morphological observation of strain MZT7. (A) Colony morphology, (B) Gram stain picture.

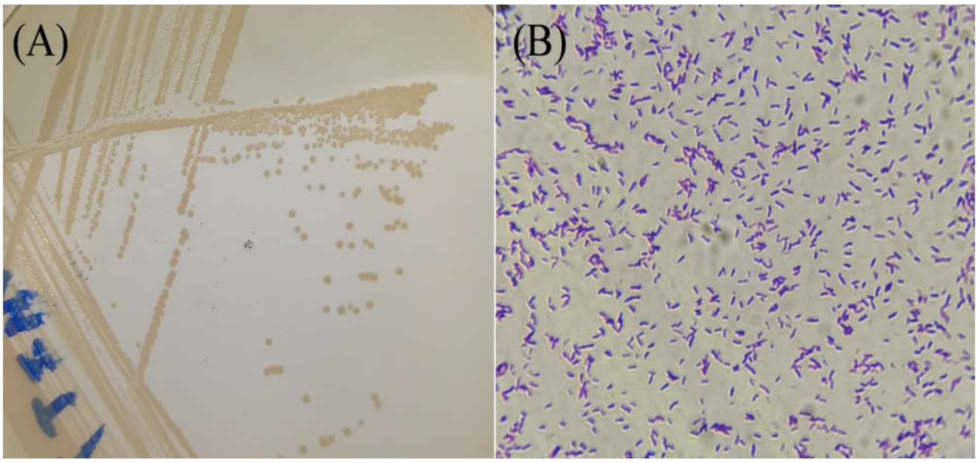

Figure S2. The circular map of the strain MZT7 genome DNA.

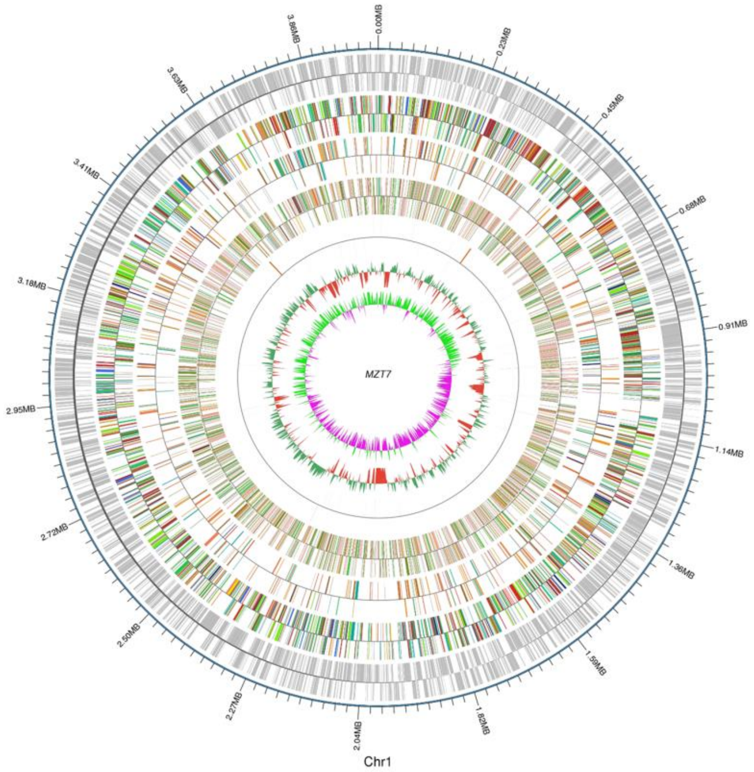

Figure S3. NR database species annotation statistics

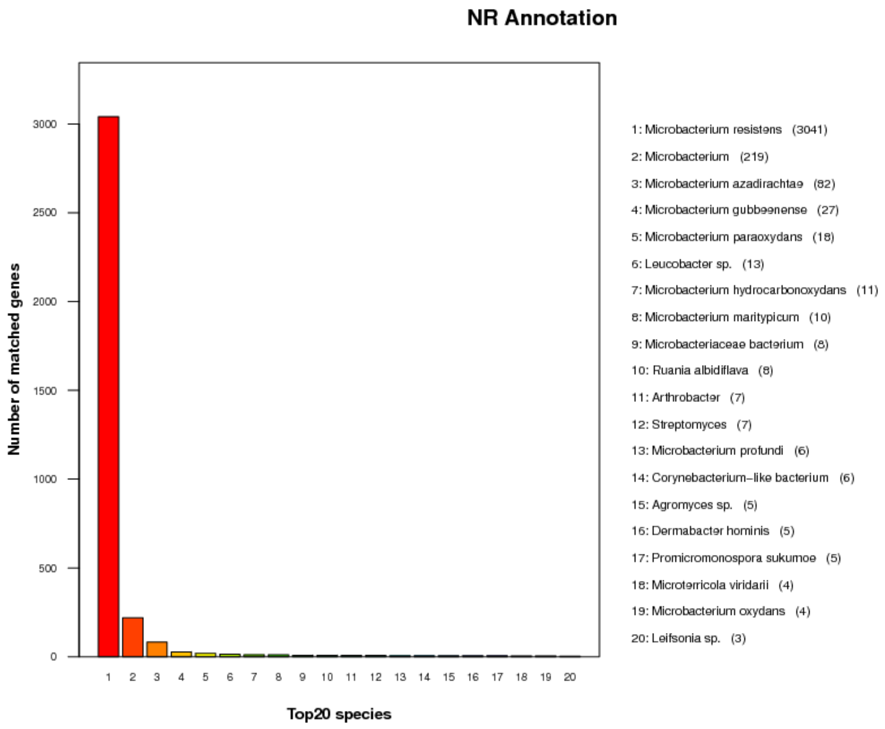

Figure S4. KEGG pathway annotation

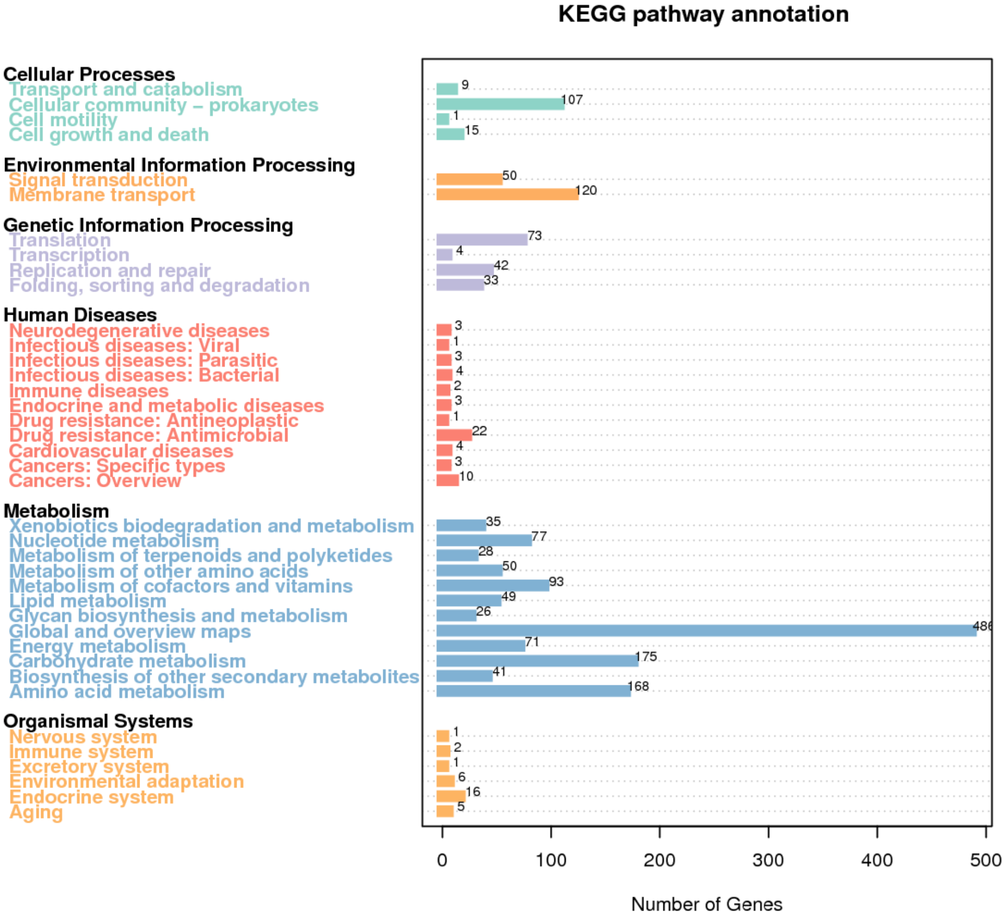

Figure S5. Base peak chromatogram. (A) Negative Mode, (B) Positive Mode.

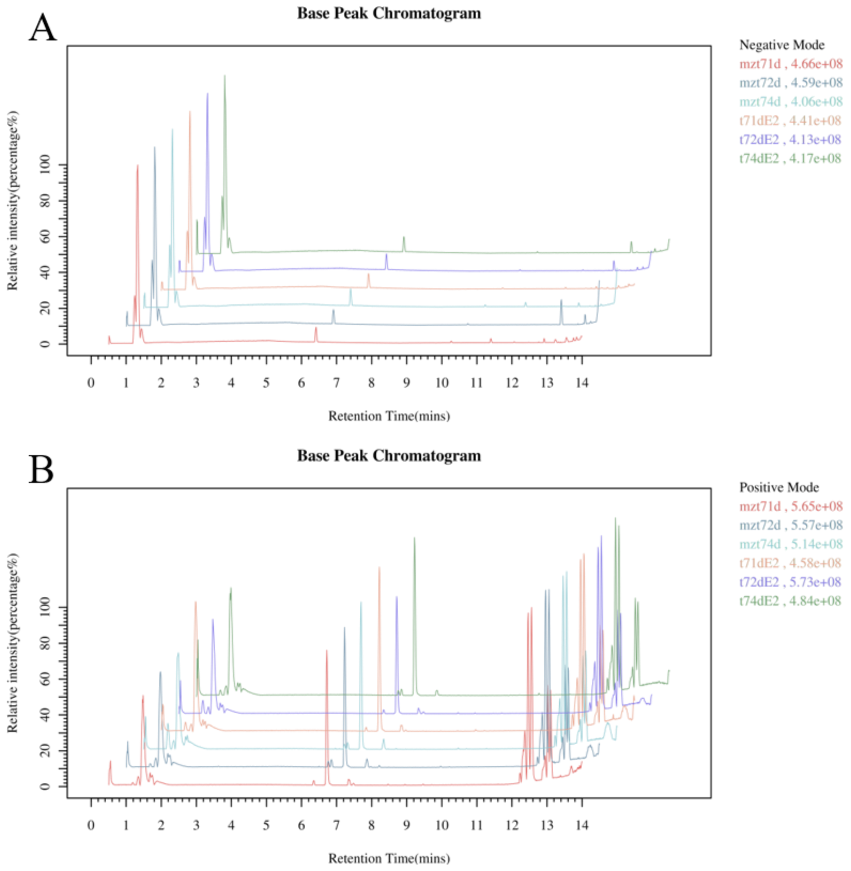

Figure S6. Mass spectra and the proposed structures of E2 metabolites.

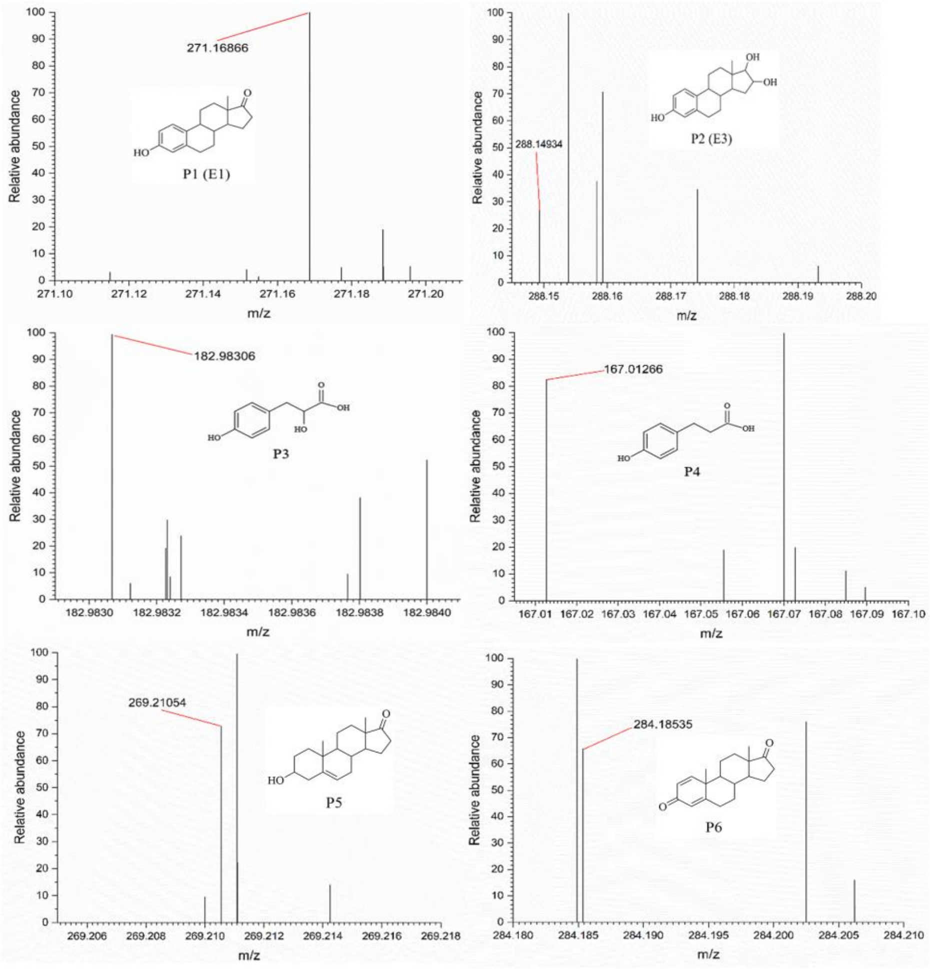

Supplement: Supplementary file 1 [file ijerph-19-11097-s001.zip › ijerph-1851456-supplementary.pdf]
